# Retinal Information is Independently Associated with Cardiovascular Disease in Patients with Type 2 diabetes

**DOI:** 10.1038/srep19053

**Published:** 2016-01-12

**Authors:** Vivian Yawei Guo, Juliana Chung Ngor Chan, Harriet Chung, Risa Ozaki, Wingyee So, Andrea Luk, Augustine Lam, Jack Lee, Benny Chung-Ying Zee

**Affiliations:** 1 Division of Biostatistics, The Jockey Club School of Public Health and Primary Care, The Chinese University of Hong Kong, Hong Kong, China; 2 Department of Family Medicine and Primary Care, The University of Hong Kong, Hong Kong, China; 3 Department of Medicine and Therapeutics, The Chinese University of Hong Kong, The Prince of Wales Hospital, Hong Kong, China; 4 Hong Kong Institute of Diabetes and Obesity, The Chinese University of Hong Kong, The Prince of Wales Hospital, Hong Kong, China; 5 Asia Diabetes Foundation, The Chinese University of Hong Kong, The Prince of Wales Hospital, Hong Kong, China; 6 Department of Family Medicine, New Territories East Cluster, Hospital Authority (Ma On Shan Family Medicine Centre), Hong Kong, China; 7 Clinical Trials and Biostatistics Lab, Shenzhen Research Institute, The Chinese University of Hong Kong, Shenzhen, China

## Abstract

To evaluate the association between a series of retinal information and cardiovascular disease (CVD) and to evaluate whether this association is independent of traditional CVD risk factors in type 2 diabetes patients, we undertook an age-sex matched case-control study with 79 CVD cases and 150 non-CVD controls. All the participants underwent standardized physical examinations and retinal imaging. Retinal information was extracted from the retinal images using a semi-automatic computer program. Three stepwise logistic regression models were evaluated: model 1 with cardiovascular risk factors only; model 2 with retinal information only and model 3 with both cardiovascular risk factors and retinal information. The areas under the receiver operating characteristic curves (AUCs) were used to compare the performances of different models. Results showed that the AUCs were 0.692 (95%CI: 0.622−0.761) and 0.661 (95%CI: 0.588−0.735) for model 1 and model 2, respectively. In addition, model 3 had an AUC of 0.775 (95%CI: 0.716−0.834). Compared to the previous two models, the AUC of model 3 increased significantly (p < 0.05 in both comparisons). In conclusion, retinal information is independently associated with CVD in type 2 diabetes. Further work is needed to validate the translational value of applying retinal imaging analysis into clinical practice.

Cardiovascular disease (CVD) is a major macrovascular complication in diabetes and the leading cause of mortality worldwide. It causes 17.3 million deaths per year, a number that is expected to project to over 23.6 million by 2030^1^. Traditional risk factors of CVD include hypertension, kidney disease, elevated levels of total cholesterol and blood sugar^2^,^3^,^4^,^5^. Nevertheless, traditional risk factors are insufficient to identify or predict all the CVD events^6^, which means that other factors could also explain part of the CVD risk.

Retinal vessels are the only vessels that can be directly seen in the human body and provide rich information, including vascular diameter, attenuation, geometry at the branching and measures reflecting how effectively the vascular network fills the retinal space. Recently, the information obtained from retinal vessels has been linked to CVD^7^,^8^,^9^,^10^. The Atherosclerosis Risk in Communities (ARIC) Study in the general population showed that narrower retinal arterioles, wider retinal venules and the presence of focal arteriolar narrowing were predictive of lacunar stroke after 11.2 years of follow-up^7^. The Blue Mountain Eye Study showed that participants with the lowest and highest quartiles of fractal dimension (FD), a dimensionless measure reflecting the complexity and density of the retinal vasculature, had a 50% higher risk of coronary heart disease mortality during 14 years of follow-up than those with optimal FD (middle quartiles)^8^.

However, inconsistent findings were found between other retinal vascular parameters with CVD. For example, a cross-sectional study showed that both retinal arteriolar and venular tortuosity were positively associated with stroke prevalence^10^. Yet, no association could be demonstrated in a follow-up study^11^. Furthermore, most studies investigating the association between retinal vascular parameters and CVD were conducted in the general population^7^,^8^,^9^,^10^,^11^, rather than diabetic patients^12^,^13^,^14^,^15^, a group with higher risk of developing CVD (2−5 times) than the general population due to the clustering of risk factors^16^. In addition, most studies only looked at a single retinal vascular parameter^7^,^8^,^9^, rather than a combination of various retinal information^10^,^11^. Therefore, in this study, we attempt to assess the association between a spectrum of retinal information and CVD among type 2 diabetes patients, and evaluate whether this association is independent of traditional cardiovascular risk factors. Our findings should add fuel to the ongoing research on the association between retinal microvascular characteristics and cardiovascular risk.

## Results

### Comparison of clinical and retinal characteristics

Among the eligible patients, there were 79 patients with CVD (20 patients only had stroke, 54 patients only had coronary heart disease, and 5 patients had both stroke and coronary heart disease). We further identified 150 age-sex matched controls from the recruited patients. Compared to the controls, CVD patients had longer diabetes duration (median years [inter-quartile range (IQR)]: 8 [4−13] vs. 4 [1−9] years, p < 0.001). They were more likely to have hypertension (92.4% vs. 79.3%, p = 0.011) and increased level of HbA1c (mean [standard deviation (SD)]: 7.3% [1.4] vs. 6.8% [1.4], p = 0.024). Other clinical factors were similar in patients with CVD and those without (Table 1).

In terms of retinal characteristics, CVD patients were more likely to have diabetic retinopathy (DR) (38.0% vs. 25.3%, p = 0.047) and smaller arteriolar-to-venular diameter ratio (AVR) (0.70 [0.04] vs. 0.72 [0.05], p = 0.006) when compared to non-CVD controls (Table 2). CVD patients had less complex retinal arteriolar branching pattern (arteriolar FD: 1.15 [0.05] vs. 1.16 [0.04], p = 0.028). Furthermore, larger arteriolar branching coefficient (BCa) (1.67 [0.23] vs. 1.60 [0.24], p = 0.042) and smaller of arteriolar junctional exponent (JEa) (−0.66 [0.23] vs. −0.54 [0.26], p = 0.001) were observed in CVD patients.

### Models using different strategies

**Figure 1:**
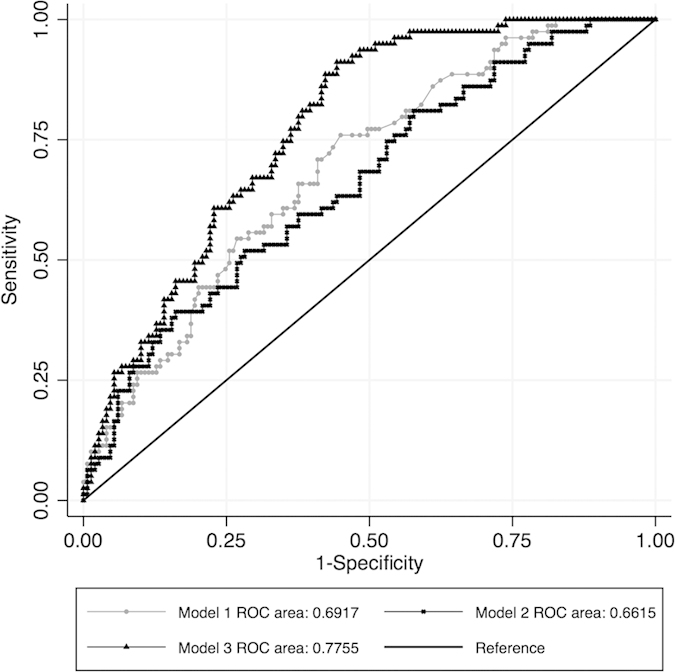
AUC comparison between different models. (Model 1: Inclusion of traditional cardiovascular risk factors only; Model 2: Inclusion of retinal information only; Model 3: Inclusion of traditional cardiovascular risk factors + retinal information).

In the model only using traditional cardiovascular risk factors (model 1), hypertension, longer diabetes duration and decreased high-density lipoprotein (HDL)-cholesterol were associated with CVD (Table 3). The area under the receiver-operating characteristic curve (AUC) was 0.692 (95%CI: 0.622−0.761) (Table 3, Fig. 1). In the model using retinal information alone (model 2), patients with DR, smaller AVR and arteriolar JE were more likely to have CVD, and the model had an AUC of 0.661 (95%CI: 0.588−0.735). Furthermore, in the comprehensive model using both traditional cardiovascular risk factors and retinal characteristics (model 3), hypertension, longer diabetes duration, higher HbA1c level, smaller AVR, arteriolar BC and JE, as well as larger venular length-to-diameter ratio (LDR) were associated with CVD. The AUC for the comprehensive model was 0.775 (95%CI: 0.716−0.834). Compared to the previous two models, the AUC of model 3 increased significantly (model 3 vs. model 1, p = 0.010; model 3 vs. model 2, p = 0.002) (Table 4). No significant difference was found between model 1 and model 2 (p = 0.544).

## Discussion:

Using an age-sex matched case-control model, our study first shows that a series of vascular information, such as vascular diameter and bifurcation-related parameters, is associated with CVD in type 2 diabetes patients. The association was independent of traditional cardiovascular risk factors. These results implied that in type 2 diabetes, assessment of retinal vasculature may have the potential to provide more information in risk estimation of CVD. However, longitudinal studies are needed to further confirm the findings in our study.

In diabetes patients, presence of DR is a marker of increased CVD risk in type 2 diabetes; this has been demonstrated in several studies^17^,^18^,^19^,^20^. In ARIC among type 2 diabetes patients, DR was an independent risk factor for incident stroke (relative risk [RR]: 2.34, 95%CI: 1.13−4.86), heart failure (RR: 2.71, 95%CI: 1.46–5.05) and coronary heart disease (RR: 2.07, 95%CI: 1.38−3.11) after multivariate adjustment of cardiovascular risk factors^17^,^18^,^19^. The severity of DR was also positively associated with the incidence of cardiovascular events^20^. Our findings suggest a positive association between DR and CVD as well.

In terms of the association between retinal vascular parameters and CVD, the Wisconsin Epidemiologic Study of Diabetic Retinopathy (WESDR) in type 2 diabetes patients did show that smaller retinal arteriolar caliber or larger retinal venular caliber were more likely to be associated with increased stroke mortality after 22-years of follow-up^12^. No significant association was found between retinal vascular diameter and mortality of ischemic heart disease in the same study^12^. Another WESDR study conducted in type 1 diabetes patients showed that smaller AVR was associated with increased risk of myocardial infarction, but not angina or stroke^13^. The Pittsburgh Epidemiology of Diabetes Complications study revealed a positive association between smaller CRAE and incidence of coronary heart disease among female type 1 diabetes patients^14^. Similar findings were also demonstrated in an African American population with type 1 diabetes, where smaller CRAE was associated with increased 6-year incidence of CVD^15^. We also found that retinal diameter-related parameters were associated with CVD. We further demonstrated that retinal bifurcation related parameters were associated with CVD in type 2 diabetes patients. However, all the studies in the same area were conducted in the general population^8^,^10^,^11^ and no comparable study focused on diabetes population. It might be because the accurate and reliable detection of complex retinal vascular parameters, such as vascular tortuosity, FD or bifurcation-related parameters, was only available recent years with the advancement of digital retinal imaging technology and computer program^21^.

Our findings, reported in this study, are not fully consistent with previously published studies^8^,^9^,^11^, which showed that retinal vascular fractal dimension, but not tortuosity or bifurcation angle, was related to CVD in the general population. Our study, however, shows that bifurcation-related parameters are significantly associated with CVD in type 2 diabetes. One possible explanation might be that different statistical methods were used in analyzing retinal vascular characteristics. Most of the previous studies assessed the association based on per quartile or per SD change of these continuous retinal vascular characteristics^7^,^8^,^9^,^10^,^12^,^22^. A second possible reason might be that different study populations were not comparable, with most studies conducted in the general population^7^,^8^,^9^,^10^,^11^,^22^ and only a few studies focused on diabetes patients^12^. Third, we included a wide spectrum of retinal vascular characteristics and investigated the overall association with CVD, while most previous studies only focused on one particular characteristic^7^,^8^,^9^,^10^,^12^,^22^. Last, our study randomly selected one eye to extract the vascular information, while some other studies measured all retinal vascular parameters exclusively from right eyes^8^ or left eyes^9^, or used the mean value from both eyes^12^,^13^,^15^. Although the left and the right eyes were substantially correlated^23^,^24^,^25^, there were still some differences caused by the different intrinsic ocular factors and non-simultaneous retinal image taking time, which may lead to the differences observed between eyes^25^,^26^. Our results add further knowledge to the ongoing debate of the association between retinal information and CVD.

The association between retinal information and CVD has several possible underlying biological mechanisms. First, there is a close connection of retinal and cerebral microvasculature during the embryonic development and they share similar vascular regulatory processes, which imply that retinal vessels may alter with changes of the cerebral vasculature^27^,^28^,^29^,^30^. Second, according to the Murray principle of minimum work, the human circulatory systems are optimized to achieve the fastest transportation rates with the minimal energy costs across any vascular network^31^,^32^,^33^. Under disease conditions, both retinal and cerebral microcirculations are affected at the same time. The vascular topography has to change in both systems in order to meet the new physiological condition. Thus, changes of retinal vessels may parallel the vascular changes in the cerebral circulation.

To our knowledge, this is the first study that considers the association between a combination of various retinal information and CVD. We found one study using retinal imaging analysis that looked at a series of retinal vascular parameters and Alzheimer’s disease^34^, and demonstrated the clinical usefulness of various retinal information. We discussed the possible underlying biological and physiological mechanisms, and the findings in our study clearly indicated an independent association of retinal information and CVD, despite the current absence of a comparable study. We are aware of the necessity of more investigations before we can promote the comprehensive retinal imaging analysis with retinal vascular measures into clinical practice. First of all, the additional significant value of various retinal parameters for CVD prediction must be demonstrated versus relying solely on the traditional cardiovascular risk factors. A study in 9,155 non-diabetic participants showed that retinal vascular diameter provides only slightly superior prediction value to coronary heart disease incidence when compared to the Framingham risk model (AUC increased from 0.695 to 0.706)^22^. Thus, whether adding more retinal information into the risk evaluation model improves the model performance still requires further investigations. Second, automatic vessel tracking technique is needed to reduce the observer input and increase efficiency. Numerous studies are now focusing on isolating the information from retinal images by automatic computer programs^35^,^36^,^37^. Retinal imaging analysis with various retinal vascular measures may soon be clinically available and provide real-time information of an individual’s potential, future CVD risk^38^. But large population studies are urgently needed to understand the normal ranges of the retinal vascular parameters.

The strengths of our study include using a reliable program to measure a wide spectrum of retinal vascular parameters^21^. We also separate retinal arterioles and venules in the analysis, as they may have different optimal microcirculation characteristics, due to the differences of physiological function between arterioles and venules^39^. Another strength is that we used the same questionnaires, with standardized protocol of clinical characteristics measurements, and the same laboratory tests. Furthermore, two trained endocrinologists graded each retinal image separately and DR was confirmed only if they agreed with each other on these results; this guaranteed the reliability of the DR grading in this study. The following limitations should also be addressed: first, this is an age-sex matched case-control study with a small sample size. The study settings precluded us from exploring the casual relationship between retinal vascular parameters and CVD. A prospective cohort study with larger sample size is needed to confirm the findings in our study and further evaluate the value of using retinal imaging as a tool to predict CVD. Second, we failed to consider the confounding effect of other cardiovascular risk factors on changes of retinal vessel parameters due to the stepwise logistic regression analysis. Last, we only considered Chinese type 2 diabetes patients. The results may not be applicable to other ethnic populations^40^.

In conclusion, we observed that retinal characteristics were associated with CVD by conducting a case-control study in type 2 diabetes patients. Our results implied that comprehensive retinal imaging analysis with retinal vascular measures may provide additional information to identify CVD. However, one still needs to define “the norm” values of these retinal parameters in the general population in order to aid the understanding in clinical settings. Also, further studies are needed to validate the translational value of retinal imaging analysis in clinical practice.

## Methods:

### Patient enrollment and study design

The study, together with all experimental protocols, was approved by the Joint Chinese University of Hong Kong – New Territories East Cluster Clinical Research Ethics Committee (CREC) and was endorsed by the Jockey Club School of Public Health and Primary Care of the Chinese University of Hong Kong. The study was performed in accordance with the approved guidelines of CREC and informed consent form was obtained from all participants. We consecutively recruited type 2 diabetes patients from January 2008 to May 2010 at Ma On Shan Family Medicine Center. Eligible participants were those aged over 18 years with diagnosed type 2 diabetes. Female patients who were pregnant or lactating, or patients with type 1 diabetes defined as presentation with diabetic ketoacidosis, unprovoked ketosis, or requirement of insulin within 12 months of diagnosis were excluded. A total of 644 Chinese type 2 diabetes patients were recruited. Every patient underwent comprehensive assessments, including questionnaires of demographic information and medical history, blood and urine tests, as well as retinal images.

Patients with CVD (1. stroke or transient ischemic attack; 2. coronary heart disease) were defined as cases, and patients without CVD were defined as controls. Medical history of CVD was first self-reported through questionnaires by the patients. Then, the CVD history of every patient was cross-checked with records from the electronic Clinical Management System (CMS) in Hong Kong or from the referring physician^41^. The CMS captures admission information of all public hospitals and it is the sole portal for information used in all public clinical settings^41^. Age and sex matched controls without medical history of CVD were selected at a ratio of around 1:2.

### Traditional risk factors measurements

Demographic information was collected using questionnaires. Smoking habits were recorded and encoded as current or non-current smokers. Blood pressure (BP) was measured from both arms after at least 5 minutes of resting and the average value was used in the analysis. Hypertension was defined as known hypertension, and/or systolic BP/diastolic BP ≥ 130/80 mmHg with/without anti-hypertension treatment.

Blood and urine samples were collected after overnight fasting to assess the level of blood glucose, glycated haemoglobin (HbA1c), lipid profile (total cholesterol, triglycerides, HDL-cholesterol and calculated low density lipoprotein [LDL]-cholesterol) and renal functions. Estimated glomerular filtration rate (eGFR) was calculated based on age, gender and serum creatinine level^42^. A spot urine sample was used to estimate urine albumin to creatinine ratio (ACR). One patient without complete clinical information was excluded from the analysis.

### Retinal information assessment

Digital fundus photographs were acquired by a trained technician using a Topcon non-mydriatic retinal camera of both eyes respectively (TRC-NW6S, Tokyo Optical Co, Tokyo). The spatial resolution of each image was 2896 by 1944 pixels and the images were stored without compression before analysis. DR was graded by two trained endocrinologists according to the International Diabetic Retinopathy Disease Severity Scale^43^. The participant was defined as having DR if either eye had hemorrhage, hard exudates, cotton wool spots or any other signs of DR^43^.

As retinal image with blurred optic disc edge or small retinal vessels may not be reliably and accurately measured, we excluded patients without acceptable quality of retinal images (n = 78) from the analysis. Then, a total of 21 retinal vascular characteristics were extracted from a randomly selected eye with a commercially available semi-automatic computer program - Singapore I Vessel Assessment program^21^. The detailed information on retinal vessel measurements was published earlier^21^. In general, retinal arterioles and venules were separated in the analysis with a lowercase ‘a’ or ‘v’ at the end of the parameter name, indicating arteriolar or venular measurements. Retinal diameter was presented as central retinal arteriolar equivalent (CRAE) and central retinal venular equivalent (CRVE) using the Knudtson-Parr-Hubbard formula from the largest six arterioles and venules, respectively^44^. The AVR was defined as the ratio of CRAE to CRVE. Standard deviation of the vessel width (BSTD) was used to indicate the difference of the diameters of the included vessels. The length-to-diameter ratio (LDR) was calculated as the ratio of vessel length from the midpoint of one vascular bifurcation to the midpoint of the next bifurcation, to the diameter of the parent vessel at the first bifurcation^45^. It reflects the diameter change independent of refractive magnification power of the eye. The complexity of the retinal vascular network was assessed using fractal dimension (FD)^46^. Vessel curvature tortuosity (TORT) was defined as the ratio of the integral of the total squared curvature of the vessel segment to the length of the vessel arc^47^. A series of bifurcation-related parameters were also extracted from retinal images. Branching angle (BA) was denoted as the angle between two daughter vessels^48^. Branching coefficient (BC) was defined as (d_1_ + d_2_)^2^/d_0_^2^, where d_0_ was the width of the trunk vessel and d_1_ and d_2_ were the two branching vessels^48^,^49^. Asymmetry factor (AF) was calculated as d_1_^2^/d_2_^2^, where d_1_ is greater than or equal to d_2_. Angular asymmetry (AA) was defined as the difference between two daughter angles of a bifurcation. Junctional exponent (JE) was defined as JE = (d_0_^3^ − d_1_^3^ − d_2_^3^)^1/3^/d_0._ These parameters describe the deviation from optimality at a bifurcation.

### Statistical analysis

The differences of clinical and retinal characteristics between CVD and non-CVD patients were compared using independent two-sample T test or Wilcoxon rank-sum test for continuous data and χ^2^ test for categorical variables. All data were expressed as mean ± SD or median (IQR) or percentages, as appropriate.

To evaluate the association between retinal information and CVD in type 2 diabetes patients and to assess whether the association is independent of the traditional risk factors of CVD, three stepwise logistic regression analyses were applied: Model 1: using traditional cardiovascular risk factors only^50^,^51^,^52^, namely HbA1c, diabetes duration, current smoking status, BP, total cholesterol, HDL-cholesterol, urine ACR and eGFR; Model 2: using retinal information only, including DR and 21 retinal vessel-related parameters mentioned above; Model 3: using both traditional cardiovascular risk factors and retinal information. All the covariates with a p value less than 0.1 were kept in the final model. To compare the performances of different models, the AUCs were compared using Delong method^53^.

All the statistical procedures were analyzed using Stata/SE (StataCorp LP, Release 12.1, College Station, TX, USA).

## Additional Information

**How to cite this article**: Guo, V.Y. *et al.* Retinal Information is Independently Associated with Cardiovascular Disease in Patients with Type 2 diabetes. *Sci. Rep.* **6**, 19053; doi: 10.1038/srep19053 (2016).

## Figures and Tables

**Table 1:** Comparison of clinical characteristics between CVD and non-CVD participants with type 2 diabetes.

Parameter	CVD	Non-CVD	p value
Number	79	150	
Age (years)	62.7 (8.3)	62.0 (7.9)	0.480
Female, n (%)	16 (20.3%)	31 (20.7%)	0.983
Diabetes duration (years)^a^	8 (4 – 13)	4 (1 – 9)	< 0.001
HbA1c (%)	7.3 (1.4)	6.8 (1.4)	0.024
Current smoker n (%)	5 (6.3%)	19 (12.7%)	0.315
Hypertension, n (%)	73 (92.4%)	119 (79.3%)	0.011
Systolic BP (mmHg)	132.6 (17.7)	133.3 (18.2)	0.794
Diastolic BP (mmHg)	75.3 (8.6)	76.8 (8.3)	0.213
Spot urine ACR (mg/mmol)^a^	1.5 (0.4−10.1)	1.1 (0.4−3.2)	0.404
eGFR (ml/min/1.73 m^2^)	98.6 (23.3)	103.1 (22.5)	0.156
Total cholesterol (mmol/L)	4.2 (1.0)	4.4 (0.9)	0.201
Triglycerides (mmol/L)^a^	1.4 (1.0−1.9)	1.3 (1.0−1.8)	0.772
HDL-cholesterol (mmol/L)	1.1 (0.3)	1.2 (0.3)	0.048
LDL-cholesterol (mmol/L)	2.3 (0.9)	2.4 (0.8)	0.203

All data were expressed as mean ± SD or median (IQR) or number (percentages), as appropriate.

The differences between CVD and non-CVD patients were compared with independent two-sample T test for continuous variables and χ^2^ test for categorical variables, unless otherwise indicated. ^a^Comparison was made with Wilcoxon rank-sum test.

Abbreviations: ACR: albumin to creatinine ratio; BP: blood pressure; CVD: cardiovascular disease; eGFR: estimated glomerular filtration rate; HbA1c: glycated haemoglobin; HDL: high-density lipoprotein; LDL: low-density lipoprotein.

**Table 2:** Retinal information comparison between CVD and non-CVD patients.

Parameter	CVD	Non- CVD	p value
Number	79	150	
DR	30 (38.0%)	38 (25.3%)	0.047
CRAE	138.94 (11.44)	140.39 (12.86)	0.401
CRVE	195.13 (15.98)	193.14 (14.82)	0.348
AVR	0.70 (0.04)	0.72 (0.05)	0.006
BSTDa	10.19 (1.92)	9.79 (1.78)	0.117
BSTDv	9.02 (1.46)	8.99 (1.71)	0.902
FDa	1.15 (0.05)	1.16 (0.04)	0.028
FDv	1.17 (0.04)	1.17 (0.04)	0.746
TORTa ( × 10^5^)	7.26 (1.12)	7.15 (0.98)	0.459
TORTv ( × 10^5^)	8.46 (1.26)	8.22 (1.04)	0.120
BAa	78.64 (10.83)	79.34 (8.62)	0.683
BAv	80.27 (8.46)	79.52 (7.78)	0.498
BCa	1.67 (0.23)	1.60 (0.24)	0.042
BCv	1.44 (0.22)	1.40 (0.20)	0.162
AFa	0.82 (0.05)	0.82 (0.05)	0.729
AFv	0.75 (0.06)	0.75 (0.07)	0.997
JEa	−0.66 (0.23)	−0.54 (0.26)	0.001
JEv	−0.33 (0.30)	−0.28 (0.27)	0.226
AAa	32.67 (10.67)	32.64 (9.81)	0.996
AAv	42.10 (9.78)	40.87 (10.45)	0.535
LDRa	9.94 (6.40)	10.85 (6.89)	0.387
LDRv	11.97 (5.69)	11.21 (5.46)	0.345

All data were expressed as mean ± SD or number (percentages), as appropriate.

The differences between CVD and non-CVD patients were compared with independent two-sample T test for continuous variables and χ^2^ test for categorical variables.

Abbreviations: AA: angular asymmetry; AF: asymmetry factor; AVR: arteriolar-to-venular diameter ratio; BA: branching angle; BC: branching coefficient; BSTD: standard deviation of the vessel width; CRAE: central retinal arteriolar equivalent; CRVE: central retinal venular equivalent; DR: diabetic retinopathy; FD: fractal dimension; JE: junctional exponent; LDR: length-to-diameter ratio; TORT: curvature tortuosity. A lowercase “a” or “v” at the end of the parameter name indicates arteriolar or venular measurements.

**Table 3:** Stepwise logistic regression analysis showing factors associated with CVD in different models.

Included parameter	Odds ratio	95%CI	p value
**Model 1: Inclusion of traditional cardiovascular risk factors only**			
Diabetes duration	1.09	1.04–1.14	< 0.001
Hypertension	2.63	1.02–6.78	0.46
HDL-cholesterol	0.39	0.14–1.09	0.72
Hosmer- Lemeshow *p*-value: 0.490; AUC: 0.692 (95%CI: 0.622–0.761)			
**Model 2: Inclusion of retinal characteristics only**			
DR	1.79	0.92–3.11	0.091
AVR	0.002	0.00–1.40	0.063
JEa	0.19	0.06–0.62	0.006
Hosmer- Lemeshow *p*-value: 0.672; AUC: 0.661 (95%CI: 0.588–0.735)			
**Model 3: Inclusion of traditional cardiovascular risk factors and retinal characteristics**			
Diabetes duration	1.08	1.03–1.14	0.001
Hypertension	2.47	0.90–6.75	0.077
HbA1c	1.29	1.02–1.61	0.031
AVR	0.002	0.00–1.94	0.080
BCa	0.10	0.01–1.06	0.056
JEa	0.03	0.003–0.33	0.004
LDRv	1.06	1.00–1.12	0.057
Hosmer- Lemeshow *p*-value: 0.267; AUC: 0.775 (95%CI: 0.716–0.834)			

Abbreviations: AVR: arteriolar-to-venular diameter ratio; AUC: area under receiver operating characteristic curves; BC: branching coefficient; DR: diabetic retinopathy; HbA1c: glycated haemoglobin; HDL: high density lipoprotein; JE: junctional exponent; LDR: length-to-diameter ratio. A lowercase “a” or “v” at the end of the parameter name indicates arteriolar or venular measurements.

**Table 4:** Comparison of area under receiver operating characteristic curves (AUC) between different models using DeLong method.

Model comparison	p value
Model 1 vs Model 2	0.544
Model 1 vs Model 3	0.010
Model 2 vs Model 3	0.002

Model 1: Inclusion of traditional cardiovascular risk factors only.

Model 2: Inclusion of retinal information only.

Model 3: Inclusion of traditional cardiovascular risk factors + retinal information.
